# Respiratory syncytial virus infection in Fischer 344 rats is attenuated by short interfering RNA against the RSV-NS1 gene

**DOI:** 10.1186/1479-0556-5-4

**Published:** 2007-02-01

**Authors:** Xiaoyuan Kong, Weidong Zhang, Richard F Lockey, Alexander Auais, Giovanni Piedimonte, Shyam S Mohapatra

**Affiliations:** 1 University of South Florida College of Medicine, Florida, USA; 2 James A Haley VA Hospital, Tampa, Florida, USA; 3 Batchelor Children's Institute, University of Miami, Miami, Florida, USA; 4 West Virginia University, Morgantown WV, USA

## Abstract

**Background:**

Respiratory syncytial virus (RSV) causes severe bronchiolitis and is a risk factor for asthma. Since there is no commercially available vaccine against RSV, a short interfering RNA against the RSV-NS1gene (siNS1) was developed and its potential for decreasing RSV infection and infection-associated inflammation in rats was tested.

**Methods:**

Plasmids encoding siNS1 or an unrelated siRNA were complexed with a chitosan nanoparticle delivery agent and administered intranasally. Control animals received a plasmid for a non-specific siRNA. After expression of the plasmid in lung cells for 24 hours, the rats were intranasally infected with RSV.

**Results:**

Prophylaxis with siNS1 significantly reduced lung RSV titers and airway hyperreactivity to methacholine challenge compared to the control group. Lung sections from siNS1-treated rats showed a sizable reduction in goblet cell hyperplasia and in lung infiltration by inflammatory cells, both characteristics of asthma. Also, bronchoalveolar lavage samples from siNS1-treated animals had fewer eosinophils. Treatment of rats with siNS1 prior to RSV exposure was effective in reducing virus titers in the lung and in preventing the inflammation and airway hyperresponsiveness associated with the infection that has been linked to development of asthma.

**Conclusion:**

The use of siNS1 prophylaxis may be an effective method for preventing RSV bronchiolitis and potentially reducing the later development of asthma associated with severe respiratory infections.

## Background

Respiratory syncytial virus (RSV) is the predominant cause of severe bronchiolitis and pneumonia in infants worldwide and also results in lower respiratory tract infections in immunodeficient and elderly adults [1]. Children born prematurely or with congenital heart abnormalities are at especially high risk for life-threatening respiratory infections by viruses such as RSV. Severe lower respiratory tract infection in infants can be fatal and frequently leads to costly hospital stays. RSV bronchiolitis in infancy is also a predisposing factor for the development of asthma later in life [2]. There is no effective vaccine commercially available against RSV and the relative weakness of the immune response in the target populations of infants and the elderly renders this possibility even less likely.

One of the most promising current strategies for protection against respiratory tract infection is intranasal treatment with vectors capable of generating RNAs that block viral replication. RNA interference (RNAi) is a natural defense of the innate immune system against viruses [3]. Double-stranded viral RNA produced during viral replication is recognized by the host RNAi system which cleaves it into short oligoribonucleotides, 20–30 bases long. These short interfering RNAs (siRNAs) then activate the cell's RNA cleavage machinery (the RNA interference silencing complex, or RISC) to destroy the viral RNA. The RSV genome is a single-stranded, negative-strand RNA that is copied by a viral RNA-dependent RNA polymerase into many positive-strand (sense) messages that are then translated into viral proteins. By introducing siRNAs complementary to specific viral mRNAs, double-stranded activating RNA can be generated that turns on the RISC cleavage system and destroys the viral message. This antiviral strategy has been tested successfully in cell culture and animal models against a number of human pathogens including influenza [4,5], hepatitis A and C [6,7], West Nile virus [8] and HIV [9,10]. The field of antiviral siRNA has recently been reviewed by Manjunath et al. [11].

We and others have demonstrated the effectiveness of antiviral siRNA using a BALB/c mouse model for RSV [12,13] and dengue virus infection [14]. The RSV genome encodes 10 proteins, and the first two genes transcribed generate nonstructural proteins known as NS1 and NS2 which are not part of the viral capsid. RSV has evolved a mechanism to counteract the body's interferon-inducible antivirus program and this was shown to involve NS1 and NS2 [15].

The NS1 mRNA is the first message produced during viral transcription and was chosen as the target for siRNA because it is known to inhibit the host antiviral defense system by reducing synthesis of type one interferons, IFN-α and IFN-β [16]. Loss of interferon production prevents activation of 2'–5' oligoadenylate synthase, the inducer of RNase L which degrades viral RNAs. Other antiviral enzymes blocked by NS1's inhibition of IFN-α, -β include the double-stranded RNA-dependent protein kinase and indoleamine 2,3-dioxygenase which normally reduce virus production by interfering with translation [12].

Deletion mutants of human RSV lacking NS1 or NS2 showed about 10-fold attenuated replication in Vero cells, but when intranasally inoculated into cotton rats, the NS1- or NS2-defective virus was reduced 100-fold relative to wild type [17]. A recombinant RSV deficient in NS1 was also only poorly infective in chimpanzees [18]. This suggested that the NS proteins of RSV, especially NS1, are critical for the *in vivo *replication of the virus and that RNA interference targeting these proteins should result in a significant reduction in RSV infectivity.

An integral part of siRNA therapy is the method of delivery of the interfering RNA. While naked RNA has been effectively used to turn off specific gene expression, it is thought that an RNA expression vector system may produce a more stable and long-lasting inhibition. Our lab has developed a plasmid-delivery system utilizing biocompatible nanoparticles made from the deacetylated glucosamine polymer, chitosan, a derivative of naturally occurring chitin from crustacean shells. Chitosan has been used for drug delivery and DNA complexation for a number of years [19] and has been proven safe for human use [20]. Expression plasmids for a desired gene product or siRNA can be adsorbed to the nanoparticles through interaction with the positively charged amine groups on chitosan, and the complex can be instilled intranasally into mice or other animals [21,22]. Chitosan has mucoadhesive properties that serve to target intranasally administered chitosan-plasmid nanoparticles to the lung mucosa where they may be taken up and expressed in macrophages and epithelial cells [23]. Protein expression from nanoparticle complexes has been found to persist *in vivo *for 2 to 3 weeks, and mice treated with a chitosan-interferon gamma plasmid complex were resistant to repeated RSV infections [unpublished results].

Here we report on the prophylactic effects of an siNS1 construct in preventing RSV infection in rats. Plasmids expressing anti-NS1 RNA or an unrelated sequence were complexed with chitosan nanoparticles and instilled intranasally one day prior to intranasal infection with RSV. The siNS1 treatment reduced RSV titers significantly and prevented the accompanying lung damage and airway hyperreactivity.

## Materials and methods

### Animals

Fischer 344 rats (14 weeks old) were purchased from Jackson Laboratory (Bar Harbor, ME) and maintained in a pathogen-free environment. All procedure were reviewed and approved by the University of Miami Committee on Animal Research.

### Cell lines and virus production

The HEp-2 cell line and RSV-A2 strain VR-1302 were obtained from the American Type Culture Collection (ATCC, Rockville, MD). The cell line was grown in Earle's modified Eagle's medium (EMEM), supplemented with 10% fetal bovine serum at 37°C in 5% CO_2_/95% air. For RSV production, HEp-2 cells at 60% confluence were infected with RSV at a multiplicity of infection (MOI) of 0.1 to 0.2 in OptiMEM (Invitrogen, Carlsbad, Calif.) for 2 h at 37°C. The medium was replaced with OptiMEM plus 2% FBS and the infection was allowed to progress until cytopathological effects were evident by microscopic examination. Cells were collected by scraping and centrifuged at 2100 × g for 10 min at 4°C. Supernatants were mixed with one-tenth volume of 1 M MgSO_4_, layered onto 30% glycerol in 50 mM HEPES (pH 7.5) and centrifuged in an SW 28 rotor at 24,000 × g for 3 h at 4°C. The viral pellet was gently rinsed with OptiMEM, resuspended at 4°C in 750 μl of 50 mM HEPES (pH 7.5), 0.01 M MgSO_4 _and 150 mM NaCl (filtered though 0.22 μm pore filter), aliquotted, and stored in liquid nitrogen.

### Construction of siRNA plasmids

The Ambion siRNA Target Finder and Design Tool was used to find potential siRNA targets along the NS1 sequence and the most effective one by in vitro transcription analysis was selected for use in the experiments reported here. An oligonucleotide was synthesized containing a 24 nt sequence of RSV NS1 (GGCAGCAATTCATTGAGTATGCTT) followed by an 8 nt loop sequence and the reverse complement of the NS1 target sequence. The nucleotide sequence for this oligo is 5'-GGC AGC AAT TCA TTG AGT ATG CTT CTC GAA ATA AGC ATA CTC AAT GAA TTG CTG CCT TTT TG-3'. When transcribed, the transcript forms a stem-loop structure. A second oligo was synthesized complementary to the first with restriction enzyme sites for *Apa*-1 and *Eco*-R1 at the 5' and 3' ends, respectively. The oligos were annealed and ligated into the siRNA expression vector, pSilencer 1.0-U6 (Ambion) to generate the construct, pSMWZ-1. The exact size of the siNS1 after Dicer processing was not determined. For the unrelated construct, siE7 (siRNA against the E7 protein of human papilloma virus, HPV_18_) the sequence is 5'-GAA AAC GAT GAA ATA GAT GTT CAA GAG ACA TCT ATT TCA TCG TTT TCT TTT TT-3'. To determine the possibility of off-target interactions, a BLAST search of mouse and human databases was done using the siRNA sequences and no significant homologies were found. Immunoblots using antibody specific for NS1 were done on protein isolates from transfected cells to verify knockdown of NS1 by the siRNA construct.

### Preparation of chitosan nanocomplexes and treatment with siRNA and RSV

Plasmids were complexed with chitosan nanoparticles as previously described to enhance stability and targeting of DNA to lung epithelial cells [21]. Plasmids were dissolved in 25 mM sodium sulfate and heated at 55°C for 10 min. High MW chitosan (Protasan CL113, Novamatrix, Norway) was dissolved in 25 mM sodium acetate (pH 5.5) at a concentration of 0.02% and heated at 55°C for 10 min. The plasmid solution was then mixed with an equal amount of chitosan solution (5:1, weight to weight ratio of chitosan to DNA) and vortexed at high speed for 30 sec. Fischer 344 rats (14 weeks old; n = 6 in each group) were given a single intranasal dose of 200 μg of siNS1 or control plasmid complexed with an equal amount of chitosan. After 24 h, they were intranasally inoculated with 1.4 × 10^7 ^PFU of RSV and the infection was allowed to continue for 5 days at which time the animals were euthanized. RSV titers were measured in lung homogenates.

### Measurement of airway hyperreactivity (AHR)

On day 4 of RSV infection, the AHR in response to methacholine challenge was measured by unrestrained, whole-body plethysmography (Buxco Instrument Co., Wilmington NC). Rats were placed inside Plexiglas chambers and their breathing rate and volume was monitored by pressure-sensitive tranducers inputting the amplified signals into proprietary software that had been calibrated for the experimental conditions (Buxco). After an acclimation period of about 5 min, the baseline enhanced pause (Penh), a measure of airway resistance, was determined by exposing the animals to an aerosol of saline and calculating Penh according to an algorithm developed by Buxco. Methacholine at specifically metered dose rates was then fed into the chambers and the Penh measured. The final Penh value for each rat is the average of all readings taken over a 5 minute period, and is expressed as percentage of baseline. An average of the Penh for all animals was used to compare the response of controls to siNS1-treated rats.

### RSV titer in the lung

At the end of the 5-day RSV infection period, rats were euthanized, lungs were removed and homogenized at 4°C using a Polytron (Brinkmann, Westbury, NY). Lung homogenates were centrifuged and equal amounts (based on the weight of lung homogenized) of supernatants were diluted 1:4 into EMEM culture medium. The homogenates were added to HEp-2 cells (80% confluent) and incubated at 37°C, with rocking every 15 min. After 2 h, the medium was replaced with fresh EMEM plus 10% FBS and cells were incubated for a further 16 h. The viral titer was measured by immunofluorescence using a FITC-conjugated antibody to RSV (Chemicon, Temecula, CA). Ethanol-fixed cells were stained for 30 min at 37°C and RSV-infected cells were counted by fluorescence microscopy.

### Bronchoalveolar lavage (BAL) cell differential

At the end of the 5-day infection period, the rats were euthanized and lungs were lavaged intratracheally with two washes of phosphate-buffered saline (PBS). The recovered BAL fluid was centrifuged at 700 × g for 5 min at 4°C and the cell pellet was resuspended in 200 μl of PBS. The BAL supernatants were used for cytokine analysis by ELISA (*see below*). The cell suspensions were then centrifuged onto glass slides using a Cytospin centrifuge (Shandon Instrument Co., Pittsburgh, PA) at 1000 × g for 5 min at room temperature. Aliquots of each BAL fluid sample were applied to three slides. The slides were air dried and cells were stained with a modified Wright's stain (Leukostat, Fisher Scientific, Atlanta, GA) which allows differential counting of monocytes and lymphocytes. A minimum of 300 cells per sample were counted by direct microscopic observation.

### IFN-γ and IL-4 analysis

IFN-γ and IL-4 levels were measured in BAL fluid supernatants and in rat lung homogenates (*for sample preparation, see above*) using ELISA kits according to the manufacturer's instructions (Rat Quantikine Colorimetric Sandwich ELISA Kits, R & D Systems, Minneapolis, MN).

### Staining of lung sections for CD4 and CD8 lymphocytes

Lung sections from siNS1-treated and control rats were fixed in 4% buffered paraformaldehyde then stained with PE-labeled anti-CD4 and FITC-labeled anti-CD8. Sections were examined by fluorescence microscopy and photographed.

### Statistical analysis

Values for experimental data are the average of at least two and usually three experiments and are expressed as means ± SEM (standard error of the mean). Comparisons were done using Student's *t *test and differences with *p *< 0.05 were considered significant.

## Results

### siNS1 reduces RSV infection and pulmonary pathology

Rats were given prophylactic anti-RSV treatments consisting of chitosan nanoparticles complexed with siNS1 plasmid or an unrelated plasmid instilled intranasally. Plasmid expression was allowed to proceed for 24 hours at which time the rats were infected with RSV.

**Figure 1:**
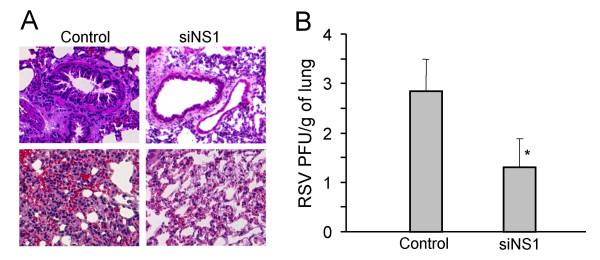
**siNS1 prevents RSV-induced lung pathology and reduces virus titer in the lung**. Rats were given intranasal treatments with chitosan-vector nanocomplexes of siNS1 or unrelated siRNA (control) followed 24 h later by intranasal inoculation with RSV. Five days later, the rats (n = 4) were sacrificed and lungs were removed, sectioned and stained for microscopic examination. The experiment was repeated once and a representative photomicrograph is shown (**A**). Another group of rats (n = 6) treated in the same way was used to determine RSV levels in the lung. Homogenates of lung tissue were assayed for RSV titer by immunofluorescence (**B**, *p < 0.05).

Animals were sacrificed five days after infection and lung sections were examined microscopically for signs of RSV-induced lung pathology. SiNS1-treated rats showed less goblet cell hyperplasia in the bronchioles (upper photos) and fewer infiltrating inflammatory cells in the interstitial regions (lower photos) compared to controls. (Fig. 1A). The number of RSV plaque-forming units in lung homogenates was also significantly decreased in rats treated with siNS1 (Fig. 1B).

### siNS1 enhances IFN-γ expression but not IL-4

**Figure 2:**
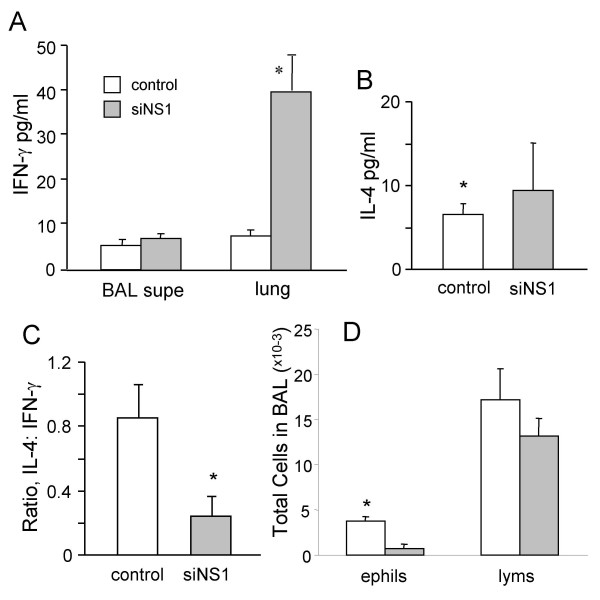
**siNS1 promotes IFN-γ production in the lung and reduces eosinophilia**. Rats (n = 6) were treated with siNS1 or control vector, then 24 h later intranasally infected with RSV. Five days later bronchoalveolar lavage was done and levels of IFN-γ and IL-4 measured in the BAL supernatants (**A-C**). BAL cells were counted and the percentage of eosinophils (*ephils*) and lymphocytes (*lyms*) was determined by differential staining (**D**). Experiments were repeated twice and values are means ± SEM (*p < 0.05).

Bronchoalveolar lavages were done on rats treated with nanoparticles carrying siNS1 or control plasmid, and IFN-γ and IL-4 were measured in BAL fluid supernatants by ELISA. The data showed that IFN-γ was significantly increased (*p *< 0.05) in siNS1-treated rat lungs (Fig. 2A) but there was no significant change in IL-4 levels between the siNS1 group and controls (Fig. 2B).

### siNS1 shifts the T lymphocyte population from Th2 to Th1

The ratio of IL-4 to IFN-γ is an indicator of the allergic phenotype in the lung–the higher the ratio, the more T helper 2-like (Th2) is the response [24]. In Fig. 2C, it can be seen that expression of siNS1 leads to a lower IL-4:IFN-γ ratio; thus a more Th1-like response to RSV infection was seen in those rats receiving treatment. In agreement with the switch from a Th2 to a Th1 phenotype, we found that siNS1 treatment substantially reduced the number of lung eosinophils (Fig. 2D). The shift from a Th2 to a Th1 type response resulting from siNS1 treatment reduces RSV-induced eosinophilia, presumably by inhibiting expression in the lung of chemokines such as eotaxin and IL-8 that attract leukocytes [25].

### Rat lung airway reactivity is decreased by siNS1

**Figure 3:**
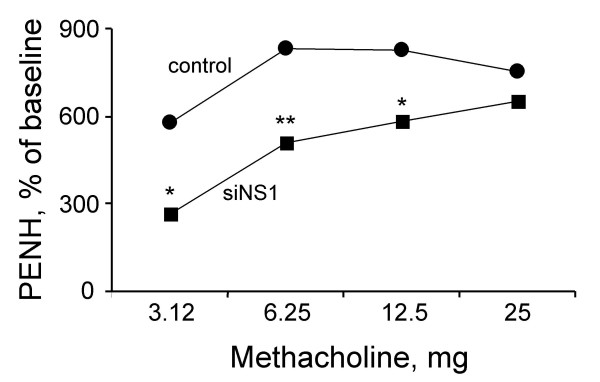
**siNS1 prevents RSV-induced airway hyperresponsiveness**. Rats (n = 4) were treated with siNS1 or control vector followed by RSV 24 h later. AHR in response to methacholine was measured 5 days later by whole-body plethysmography and the enhanced pause (Penh) was expressed as percentage of baseline (buffer only). Experiments were repeated twice. *p < 0.05, **p < 0.01.

The effect of siNS1 treatment on airway hyperreactivity (AHR) to methacholine was measured as change in the enhanced pause (Penh) relative to baseline by whole-body plethysmography. siNS1-treated rats showed significantly lower AHR (% Penh) compared to controls (Fig. 3).

### T lymphocyte infiltration in lung is altered by siNS1 treatment

**Figure 4:**
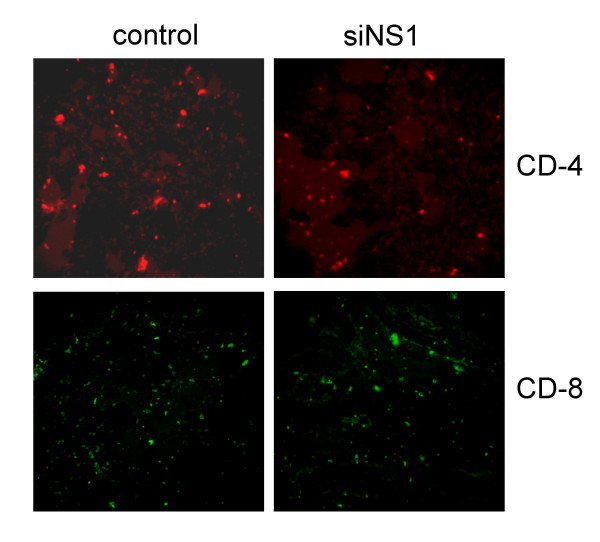
**siNS1 decreases CD4+ T cell infiltration of lung tissue**. Rats (n = 6) were given siNS1 or control vector followed by RSV 24 h later. After 5 days, the lungs were removed, sectioned and stained for CD4 and CD8. Experiments were repeated twice and representative fluorescent micrographs are shown.

RSV infection typically results in a migration of CD4+ T cells to the lung and interstitial infiltration of the surrounding tissues. Intranasal administration of siNS1 reduced the number of CD4+ T cells in the lung but did not significantly alter the infiltration of CD8+ cells (Fig. 4). The CD4+ T cell infiltration associated with RSV infection can promote clearance of the virus but at the expense of increased damage to lung epithelium and parenchyma. The decrease in CD4+ T cell numbers in the lung produced by siNS1 treatment when combined with enhanced interferon antiviral activity generates a highly effective viral clearance without damaging airway tissues.

## Discussion

RSV is a ubiquitous virus of worldwide distribution, and RSV infection is a serious health risk for bronchiolitis and pneumonia among children less than six months old. It is one of the major causes of hospitalization in that age group. The difficulty of producing an effective vaccine against RSV in infants arises from the immaturity of their immune system which is at a stage of development that precludes a full-scale immune response. Therefore, effective antiviral prophylaxis in infants must utilize a different approach. RNA interference to knock down key viral genes necessary for replication is a promising strategy for achieving such a reduction in the incidence or severity of RSV infection. In our rodent experiments, prophylactic treatment as early as three days prior to RSV infection still afforded some protection against the virus. Therapeutic siNS1 applications may also be effective in attenuating an existing RSV infection by blocking additional virus replication.

The immune response to viral infection involves specific lymphocyte proliferation and differentiation changes defined by the T helper cell phenotypes Th1 or Th2 and their associated cytokine expression. RSV infection is commonly associated with a Th1/Th2 imbalance that is shifted towards the Th2 phenotype characterized by lower IFN-γ production and increased IL-4. IFN-α is known to promote a Th1-type response [26] and the inhibition of IFN-α by the NS1 protein may be associated with this Th2 polarization. Blocking NS1 synthesis shifts the balance back to a Th1 phenotype and prevents the RSV-induced blunting of the host's immune response by enhancing IFN-γ production and thereby potentially allowing full expression of antiviral interferons and the 2',5'-oligoadenylate synthase and RNase L defense systems.

The association between CD4+ T cell infiltration and lung pathology during RSV infection has been documented [27]. Passive introduction of CD4+ T cells isolated from RSV-infected mice into naïve mice followed by intranasal administration of RSV led to severe lung damage and eosinophilia [28]. The observation that blocking the expression of the RSV NS1 protein decreased CD4+ T cell levels in the lung is an important finding that suggests NS1 may have effects on the adaptive immune system in addition to its role in subverting the innate interferon response. The interaction between regulatory T cells and effector T cells in the immune response to RSV infection may be another area of involvement of the NS proteins [29].

## Conclusion

RNA interference is being aggressively tested as a new and effective way of specifically silencing genes involved in disease pathogenesis. Currently there are three clinical trials underway to test the safety and efficacy of siRNAs–two for macular degeneration and one against RSV-induced pneumonitis. The RNA interference technique has virtually unlimited potential but its successful application will depend largely on the continued elucidation of the protein interactions and signaling pathways involved in the etiology of complex diseases such as cancer and asthma. The use of siRNA as an antiviral agent involves a relatively straightforward attack on one or more key viral genes and should be effective against many human pathogens including influenza and HIV as well as RSV. The use of non-integrating plasmid vectors obviates the risk of positional mutagenesis caused by some viral vectors, and the development of specific drug-delivery systems such as our chitosan-based nanoparticles can provide efficient targeting to infected cells. In this report, the effectiveness of RNA interference in reducing RSV infection and lung pathology was demonstrated in an animal model. It is hoped that this strategy can be implemented to stop the worldwide morbidity and mortality associated with RSV infection in infants and immunodeficient adults where vaccination is ineffective.
